# Psychometric properties of a French version of the Jefferson Scale of Empathy

**DOI:** 10.5116/ijme.62d2.8497

**Published:** 2022-07-29

**Authors:** Mariem Ghardallou, Chekib Zedini, Jihene Sahli, Thouraya Ajmi, Hedi Khairi, Ali Mtiraoui

**Affiliations:** 1 University of Sousse, Faculty of Medicine of Sousse, Department of Community Medicine, Research Laboratory LR12ES03, Tunisia; 2 University of Sousse, Faculty of Medicine of Sousse, Research Laboratory LR12ES03, Tunisia

**Keywords:** Jefferson scale of empathy, medical student, Exploratory factor analysis (EFA), Confirmatory factor analysis (CFA), reliability

## Abstract

**Objective:**

To assess the reliability and construct validity of a French version of the Jefferson Scale of Empathy-Students.

**Methods:**

A cross-sectional study was performed among undergraduate medical students in Tunisia. A total of 833 students completed a French version of the JSE-S using convenience sampling. To identify the internal consistency aspect of the reliability, Cronbach's alpha coefficient was computed. Moreover, to assess the construct validity, the sample was randomly divided into two groups. Data from the first group (n=415) were subjected to exploratory factor analysis (EFA), with principal axing factoring (PAF) and oblimin rotation, to re-examine the underlying factor structure of the scale. Data from the second group (n=419) were used for confirmatory factor analysis (CFA) to confirm its latent variable structure. Some goodness-of-fit indices were used to assess the hypothesized model. Gender groups were compared using a t-test to check the known-group validity.

**Results:**

Reliability analysis reported an acceptable level of internal consistency, with an overall Cronbach's alpha of 0.78 (95% CI [0.75,0.80]). EFA identified a two-factor structure, accounting for 27.4% of the total variance. The two-factor model produced good fit indices when item correlated errors were considered (χ^2^/df = 1.95, GFI = 0.92, CFI = 0.90, PCFI = 0.79, PGFI = 0.73 and RMSEA = 0.04). Female students had a statistically significant higher empathy scores than male students (t _(830) _= - 4.16, p < .001).

**Conclusions:**

The findings support the construct validity and reliability of a French version of the JSE for medical students. This instrument appears to be useful for investigating empathy among French-speaking populations.

## Introduction

In addition to cognitive abilities and procedural skills, students' personalities with regard to their personal qualities, attitudes, interests, values, and other psychosocial characteristics are the second pillar of medical education. Among the personality attributes, empathy is considered a significant predictor of the clinical competence of physicians-in-training and patient outcomes.^1^ According to Hojat and colleagues, empathy in patient care is defined as a predominantly cognitive attribute rather than an affective or emotional one, involving an understanding rather than a feeling of the patient's pain and suffering, combined with a capacity to communicate this understanding and an intention to help.^2^

Thus, fostering medical students' empathic skills has become one of the goals of medical education, recommended by a number of international educational councils^3^ and the World Health Organization (WHO). In fact, WHO considers empathy as a crucial skill to be endorsed in the context of medical education.^4^ To meet these international recommendations, a number of studies have focused on empathy in medical education. However, these studies continue to rely on the instruments already used. These instruments must be valid and reliable.^5^ Several instruments for measuring empathy are available and have been used in the context of medical education, such as the Interpersonal Reactivity Index (IRI) and the Emotional Empathy Scale.^6^ However, so far, only the Jefferson Scale of Empathy (JSE) has defined the meaning of empathy in the context of health professions education and patient care. Thus, this scale fits well with the need for a content-specific and context-relevant instrument for measuring empathy.^1^

Over the past twenty years, several versions of JSE have been used in different settings. Three versions of JSE are available. One version is used for medical students (S-Version). Another version was developed for practicing health professionals, including physicians, nurses, dentists, pharmacologists, clinical psychologists, and other clinicians involved in patient care (HP-Version). The third version (HPS-Version) was developed for all health professions students, other than medical students.^5^^,^^6^ All three versions are very similar in content, with minor differences in only a few words to adjust the instrument to its target population.^5^^,^^6^

In the context of medical education, using JSE-S enables medical educators "to evaluate the effectiveness of educational interventions aimed at promoting empathy".^7^ JSE-S has been widely used in different countries and cultures; however, the quality of reporting the psychometric properties of JSE-S is still sometimes suboptimal.^8^ Construct validity is an important criterion that has to be checked when assessing the methodological quality of studies investigating the measurement properties of self-reported outcome measurement tools,^9^ such as JSE. Construct validity refers to the extent to which a test measures the theoretical constructs of the attribute that has to be measured.^10^ In this regard, factor analysis of JSE-S helps to determine whether the underlying factors of the scale are consistent with the theoretical constructs of the concept measured or not (i.e., empathy among medical students in this case).^11^

In this respect, a systematic review of psychometric assessment of JSE using the COSMIN risk of bias checklist, published in 2019,^8^ reported that 22 studies out of 23 had examined the construct (or structural) validity of the JSE-S among medical students: nine of them performed a confirmatory factor analysis (CFA) and consequently received a "very good" rating, 11 studies conducted exploratory factor analysis (EFA) and were therefore considered to be "adequate", and two studies did not report any type of factor analysis and were therefore scored "inadequate".

The three-factor structure of JSE-S was the most commonly reported, with nine studies describing this arrangement.^8^ Only one study supported a four-factor structure among German medical students.^12^ However, the two-factor structure was reported by two studies conducted among students from other healthcare professions using the JSE-HPS-Version: Australian paramedical students^13^ and American pharmacy students.^14^

The aforementioned factor-analytic studies provide clues about the underlying components of the JSE-S in various samples in a variety of cultures. However, despite the accumulating evidence, it is always interesting and recommended to perform additional large scale exploratory and confirmatory factor-analytic research, using split samples from the same population to reaffirm the underlying components of JSE-S and to further confirm its latent variable structure.^6^

French is the fifth world language and the official language in 29 countries across various continents; most of these countries are members of the "Organisation Internationale de la Francophonie (OIF)". Tunisia belongs to this organization as a former French protectorate, and French is used as a second language. About 52% of the Tunisian population speaks French.^15^

This is the result of the Tunisian educational system that is set to produce bilingualism involving the French and Arabic languages. Indeed, French is taught starting from the third year of primary education and extending to secondary education in a fairly intensive way. Scientific subjects are taught in French. Thus, better bilinguals are paradoxically produced in the science streams than in the literary streams.^16^ Almost all medical students come from scientific backgrounds, with a great mastery of the French language. In addition, the entire Tunisian medical curriculum is taught in French. Therefore, the cultural diversity resulting from language and the cross-cultural characteristics of Tunisia provides an ideal ground to test the psychometric properties of a French version of JSE-S among Tunisian students. Overall, two French versions of the JSE-S were developed. The first one was designed by a Swiss team for Jefferson Medical College. However, up to now, it has not been tested in a French-speaking population. The second one was developed by a French team from the Paris Descartes College of medicine,^17^ but it was judged inadequate according to the COSMIN guidelines.^8^

Thus, the purpose of this study was to reaffirm the underlying components of the French version of JSE-S developed by the Swiss team by using exploratory factor analysis and to confirm its latent variable structure by using confirmatory factor analysis among Tunisian medical students. A further aim was to identify the reliability of this scale version.

## Methods

### Study design, participants

A cross-sectional study was conducted to assess the reliability and the construct validity of a French version of JSE-S. A convenient sample of students from the Faculty of Medicine of Sousse (Tunisia) was therefore invited to participate in this study. All students from the 1st, 2nd, 4th, and 5th academic years that consented to participate were included. This study was approved by the Research Ethics Committee of the Faculty of Medicine of Sousse. Before starting the investigation, informed consent was obtained from all respondents. Collected data were kept confidential, and responses were anonymous.

### Data collection method

The JSE-S was used to respond to the purpose of the study. This version of the questionnaire is designed to assess students' attitude towards empathy in the context of patient care. It is a self-reporting questionnaire including 20 items answered on a seven-point Likert type scale (1=strongly disagree, 7=strongly agree), and it takes five minutes to complete. Ten of the items are phrased positively and scored directly, while the other ten is phrased negatively and reverse-scored for statistical analysis.^18^ The total score was obtained by summing all the items. The possible scores range from 20 to 140. The higher the score is, the greater the participants' empathic orientation towards patient care is. The original JSE-S comprises three domains:

    ·   "Perspective Taking" (PT): it comprises ten items: 2, 4, 5, 9, 10, 13, 15, 16, 17 and 20, and it refers to the ability to analyze another person's problem from the outside.

    ·   "Compassionate care" (CC): it consists of eight items: 1, 7, 8, 11, 12, 14, 18 and 19, and it is defined by the activity in favor of the one we see suffering.

    ·   "Standing in the patient's shoes" (STS): it is composed of two items: 3 and 6, and it refers to the act of thinking as if we were in the other person's place.

A respondent must answer at least 16 of the 20 items (80%); otherwise, the questionnaire is regarded as incomplete and is therefore excluded from data analysis. Up to four blank items per respondent will be replaced with the respondent's rounded mean score, as suggested by the author of the scale.^2^ Previous investigations have supported the validity and reliability of the original as well as many translated JSE-S versions.^5^^,^^6^ In the current study, the French version of the JSE-S developed by Gerbase and colleagues from the University of Geneva (Switzerland), and researchers from the Jefferson Medical College was used. This version has never been validated among a French-speaking population.^17^ A Permission to use this French version of JSE-S was obtained from the Center for Research in Medical Education and Health Care at Jefferson Medical College. No changes were made to this version. The survey also included a set of questions to compare the mean differences in empathy scores with regard to demographic and academic characteristics, such as gender, age, and year of study.

### Procedure

Questionnaires were distributed to students during their regular academic classes (Public health, Epidemiology, Biostatistics, and research methodology). Permission to perform the survey during classes was obtained in advance from the faculty members in charge of these classes. All the students attending any of these classes were invited to participate in this study. Those who were absent on that day were not included. They were specifically informed of the study aims. They were also informed that participation is voluntary and that responses would be kept anonymous and confidential. Students willing to participate provided oral consent and completed the paper versions of the study instrument. No reward was provided for participation.

### Data analysis

Data analysis was conducted using SPSS and AMOS statistical package, with a level of significance set at p < .05. Descriptive analysis (mean (M) and standard deviation (SD)) of all the items was performed. The communalities (h^2^), which are the proportion of the variance in the variable that is accounted for by the common factors, were also estimated to give the factor structure. The corrected item-total score correlation, which is the degree to which each item correlates to the total score, was determined to identify the items that need to be revised. The total score in calculating item-total score correlation was the sum of all the items minus the particular item used in the corresponding correlation.

To assess the internal consistency of the JSE-S, Cronbach's alpha coefficient (α) was computed. It represents the degree to which all scale items measure the same construct. A coefficient alpha greater than or equal to 0.70 would be considered to show satisfactory reliability of the scale score.^19^^,^^20^

Then, to assess the construct validity of this French version, the factor structure of the JSE-S was examined through both exploratory and confirmatory factor analyses. The sample was therefore randomly divided into two groups. Data from the first group were subjected to exploratory factor analysis (EFA) to re-examine the underlying factors of the scale, and data from the second group were used for confirmatory factor analysis (CFA) to confirm its latent variable structure. EFA, as a theory-generating model, describes how and to what extent the observed variables (Items) are related to their constructs or latent variables (Factors). In the present study, it was applied in the following way. First, Bartlett's test of sphericity and Kaiser-Meyer Olkin (KMO) measure of sampling adequacy were used to verify the appropriateness of factor analysis. The KMO index ranges from 0 to 1, with 0.50 being considered suitable for factor analysis. A statistically significant Bartlett's Test of sphericity indicates that sufficient correlations exist among the variables to proceed with EFA.^21^

Secondly, Principal Axis Factoring (PAF) was selected as the factor extraction method. It was run on items 1-20 to reveal their underlying factors/constructs represented by their common variance. PFA is the most appropriate statistical procedure to achieve this purpose. Thirdly, the retained factors were submitted to a direct oblimin rotation to obtain a more interpretable simple structure. Because the eigenvalues-greater-than-one-rule (EV>1) always severely overestimates the number of components to retain and to find the best interpretable solution, the EV>1.5 rule was used to retain the number of factors in this study.^22^ In addition, factor coefficients of 0.35 or greater were required for the interpretation of the factor structure.^4^

Unlike EFA, CFA is a theory-testing model that starts with a hypothesis prior to analysis. This hypothesis can be based on theory, research, or both.^7^^,^^11^ Three models were tested in this study using CFA. The first model, Model A, is based on the three-factor structure of the original S-Version of JSE, used among American medical students.^5^ The second model, Model B, is based on the findings from EFA that were applied to the first group of the study sample. It is a model with "no correlated errors". Finally, a third model, Model C, was tested with possible violations of "no correlated errors". The structural Equation Modeling (SEM) framework was applied to confirm JSE-S latent variable structure. The regression coefficient for one item to the latent variable path for each latent variable was set to 1.0 to measure the latent variable, and covariances among the latent variables were modeled. The model parameters were estimated by using maximum likelihood. The goodness-of-fit indices resulting from this analysis are reported. They are: c^2^ and its subsequent ratio with a degree of freedom (c^2^/df), Goodness-of-fit index (GFI), Comparative Fit Index (CFI), Parsimony Comparative Fit Index (PCFI), Parsimony Goodness-of-Fit Index (PGFI), and Root Mean Square Error of Approximation (RMSEA). The model was considered to have an acceptable or good fit, respectively, if c^2^ /df was less than 5 or 2, GFI was higher than 0.9 or 0.95, CFI was higher than 0.8 or 0.9, PCFI and PGFI were higher than 0.6 or 0.8, and RMSEA was lower than 0.08 or 0.05.^19^

Moreover, gender groups were compared using a t-test to check the known-group validity.

## Results

A total of eight hundred and thirty-three of 1010 medical students enrolled in the first, second, fourth, and fifth years (82.5%) completed at least 80% of JSE-S and were, therefore, eligible for analysis. The majority of participants were females (70.3%). The students' ages ranged from 18 to 31 years, with a mean of 21.31 years (SD = 1.84).

### Item statistics

Respondents used the full range of possible answers (1-7) for each item. Item mean scores ranged from low at 2.53 (SD = 1.78) for item 18: "Physicians should not allow themselves to be influenced by strong personal bonds with their patients and their family members" to high at 6.38 (SD = 1.41) for item 2: "Patients feel better when their physicians understand their feelings."

### Item-Total Score Correlations

The corrected item-total score correlations ranged from low at 0.14 for item 3: "It is difficult for a physician to view things from patients' perspectives" to high at 0.60 for item 20: "I believe that empathy is an important therapeutic factor in medical treatment." Almost all correlations were positive and statistically significant (p < .05), except item 18 (r _(413)_ = - 0.25, p = n.s) and item 6 (r _(413) _= 0.06 , p = n.s). This finding indicates that almost all items contributed to the total score of the JSE scale. Item-total score correlations are reported in Table 1.

### Reliability analysis

Reliability analysis yielded an overall Cronbach's alpha of 0.78 (95% CI [ 0.75, 0.80]) for a sample of N=833. Reliability of each of the two factors was F1 (α = 0.79) and F2 (α = 0.67) (Table 1). Reliability coefficients of these magnitudes were considered acceptable.

### Exploratory factor analysis: Principal Axing Factoring (n= 415)

Kaiser-Meyer-Olkin analysis yielded an index of 0.86, indicating that it was appropriate to use factor analysis on this set of data. Also, the Bartlett's test of sphericity was significant (χ^2^_(190)_ = 1917.451, p < .001), showing that the inter-correlation matrix was factorable.

Analysis of the 20 items using exploratory factor analysis (EFA) with principal axis factoring (PAF) and direct oblimin rotation identified two factors with Eigen values greater than 1.5, together accounting for 27.4 % of the variance. The Eigen values for the two retained factors before rotation were 5.11 and 1.75, accounting for 22.4% and 5.0 % of the total variance, respectively. The first factor, known as "Perspective taking" in previous studies, comprised ten items with factor loadings above 0.35. The second factor included five items with factor loadings above 0.35. This factor reflected, to some extent, the second factor in the original student version labeled "Compassionate Care". The five obtained items in this factor were identical to the eight items of the original version. This factor also included the two items (Items 3 and 6) of the third factor of the original version, known as 'Standing in the Patient's Shoes". The remaining items (1, 3, 6, 18, and 19) did not significantly load in either factor (F1 or F 2) (factor loadings being less than 0.35), suggesting that these items may be inappropriate in their present form when used. A summary of the results of factor analysis for the 20 items of JSE-S are reported in Table 1.

### Confirmatory factor analysis: Structural Equation Modeling (n=418)

CFA was used to test three models. Model A was the three-factor structure found in the original JSE-S and it was tested on the total sample (N=833). Thus, the 20 items constituting the JSE-S were modeled as resulting from one of the three underling latent variables: "Perspective Taking" (10 items), "Compassionate Care "(8 items), and "Standing in the patient's shoes" (2 items). Model B was based on the findings from exploratory factor analysis. Although items 1, 3, 6, 18, and 19 of the JSE-S had low factors loading, they were not excluded from model B. Thus, all items were tested on half of the sample (n=418), and they were modeled as resulting from one of the two underlying latent variables: PT (11 Items) and CC (9 Items). It was a model with "no correlated errors". Finally, Model C was tested with possible violations of "no correlated errors" regarding the modification indices.

**Table 1:** Factor pattern coefficients, mean and SD, communalities (h2) for Principal Axing Factoring with Direct Oblimin rotation and corrected item-total correlations on the 20 items of the JSE-S (n= 415)

Item	Rotated Factor coefficients		M	SD	h^2^	r _i__-t_
	F1	F2				
10. Patients value a physician's understanding of their feelings which is therapeutic in its own right	0.61	0.18	5.74	1.61	0.46	0.55
20. I believe that empathy is an important therapeutic factor in medical treatment	0.60	0.19	5.54	1.71	0.45	0.60
9. Physicians should try to stand in their patients' shoes when providing care to them	0.55	- 0.17	4.70	1.94	0.28	0.36
17. Physicians should try to think like their patients in order to render better care	0.54	- 0.21	4.48	1.94	0.28	0.31
13. Physicians should try to understand what is going on in their patients' minds by paying attention to their non-verbal cues and body language	0.54	0.25	5.65	1.68	0.43	0.56
16. Physicians' understanding of the emotional status of their patients, as well as that of their families is one important component of the physician-patient relationship	0.53	0.25	5.89	1.52	0.42	0.54
2. Patients feel better when their physicians understand their feelings.	0.50	0.20	6.38	1.41	0.35	0.47
4. Understanding body language is as important as verbal communication in physician patient relationships	0.50	0.06	5.67	1.74	0.27	0.39
5. A physician's sense of humor contributes to a better clinical outcome	0.48	-0.74	4.94	1.93	0.21	0.33
15. Empathy is a therapeutic skill without which the physician's success is limited.	0.36	0.00	4.55	2.03	0.13	0.28
18. Physicians should not allow themselves to be influenced by strong personal bonds between their patients and their family members.	-0.29^ b^	-0.04	2.53	1.78	0.09	-0.25^*^
11. Patients' illnesses can be cured only by medical or surgical treatment; therefore, physicians' emotional ties with their patients do not have a significant influence in medical or surgical treatment.	0.20	0.58	5.69	1.74	0.44	0.49
7. Attention to patients' emotions is not important in history taking	0.22	0.55	6.11	1.59	0.42	0.51
8. Attentiveness to patients' personal experiences does not influence treatment outcomes	0.16	0.51	5.74	1.76	0.33	0.44
14. I believe that emotion has no place in the treatment of medical illness.	0.36	0.38	5.82	1.71	0.36	0.52
12. Asking patients about what is happening in their personal lives is not helpful in understanding their physical complaints	0.18	0.38	5.59	1.80	0.22	0.37
6. Because people are different, it is difficult to see things from patients' perspectives	-0.17	0.30^ b^	4.07	1.75	0.09	0.06^*^
3. It is difficult for a physician to view things from patients' perspectives.	-0.08	0.28^ b^	4.27	1.69	0.18	0.14
19. I do not enjoy reading non-medical literature or the arts	0.19	0.23^b^	5.83	1.79	0.13	0.28
1. Physicians' understanding of their patients' feelings and the feelings of their patients' families does not influence medical or surgical treatment	0.06	0.19^ b^	4.69	2.12	0.05	0.19
Eigen value	5.11	1.75				
% Variance	22.4	5.0				
Cronbach’s alpha	0.79	0.67				
95% CI	[0.76,0.82]	[0.62,0.71]				

*r _i__-t_*= Corrected item-total score correlations; ^*^Non-significant, all other item-total score correlations were statistically significant (p < .05) ; *h^2^*= communality.

Principal Axing Factoring with Oblimin rotation was used for half of the sample (n=415). Confirmatory factor analysis was performed in the other half of the sample.

^a^Items were listed by order of magnitude of the factor coefficients within each factor. Items were scored using a seven -point Likert type scale. Factor 1 was considered as a construct involving "Perspective Tacking" and Factor 2 as a construct involving "Compassionate Care". Numbers represent the sequence of the items in the actual scale.

^b^Factor coefficients < 0.35.

The fit indices of the three hypothesized models are presented in Table 2. The χ^2 ^value for the three tested models was significant, showing a poor fit between each hypothesized model and the model data. However, it is also well-known that chi-square statistics are sensitive to the sample size and that large samples can produce significant chi-square values (indicating misfit), even when the fit is acceptable.^1^^,^^5^ For this reason, a number of other model-fit indices are suggested.

Confirmatory Factor Analysis revealed acceptable fit indices values for models A (three-factor model) and B (two-factor model with no correlated errors). However, model A produced a marginally good fit with the data supporting the three-structure found in the original JSE-S (Table 2).

**Table 2:** Goodness of Fit Indices for three tested models of the JSE-S

Fit indices	Model A	Model B	Model C	Critical values Acceptable or good
χ^2^	591.56	451.644	325.14	-
df	167	169	166	-
p	< .OO1	< .OO1	< .OO1	-
χ^2^/df	3.54^*^	2.67^*^	1.95^**^	< 5 or 2
GFI	0.93^*^	0.90^*^	0.92^*^	³0.9 or 0.95
CFI	0.87^*^	0.83^*^	0.90^**^	³0.8 or 0.9
PCFI	0.77^*^	0.74^*^	0.79^*^	³0.6 or 0.8
PGFI	0.74^*^	0.72^*^	0.73^*^	³0.6 or 0.8
RMSEA	0.05	0.06	0.04^**^	<0.08 or 0.05

Model A: Three-factor structure of the original version was tested on the entire sample (N=833) Model B: Two-factor structure with "no correlated errors" (n=418) Model C: Two factor structure with "correlated errors" (n=418) *Values are acceptable; **Values are good

**Figure 1:**
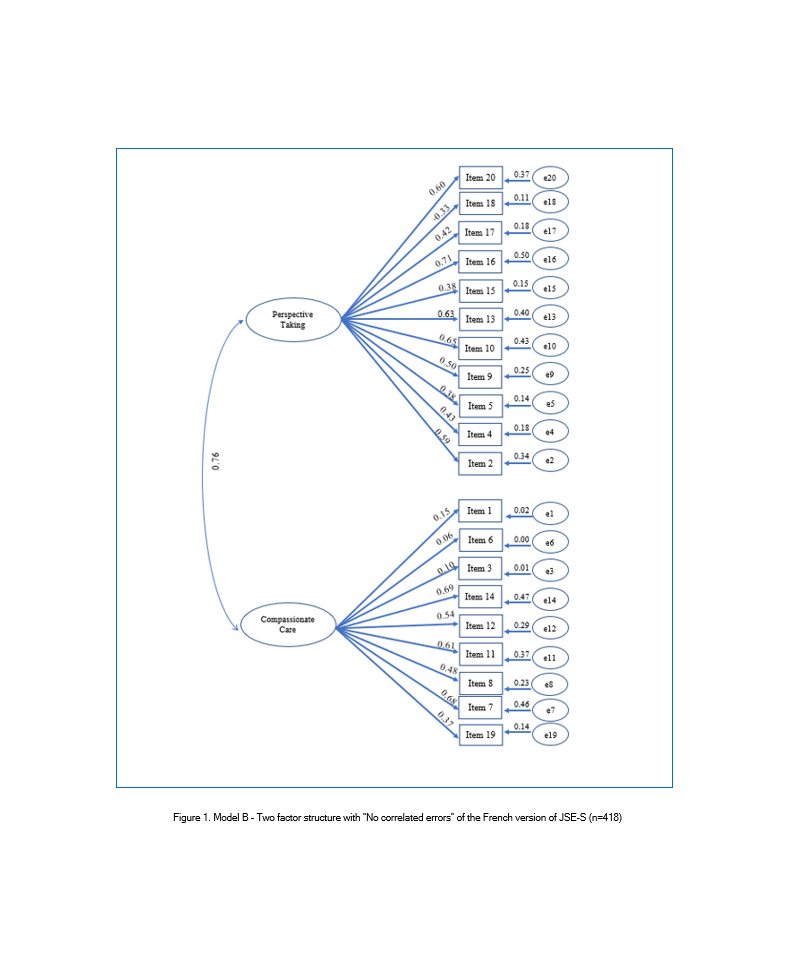
Model B - Two factor structure with “No correlated errors” of the French version of JSE-S (n=418)

**Figure 2:**
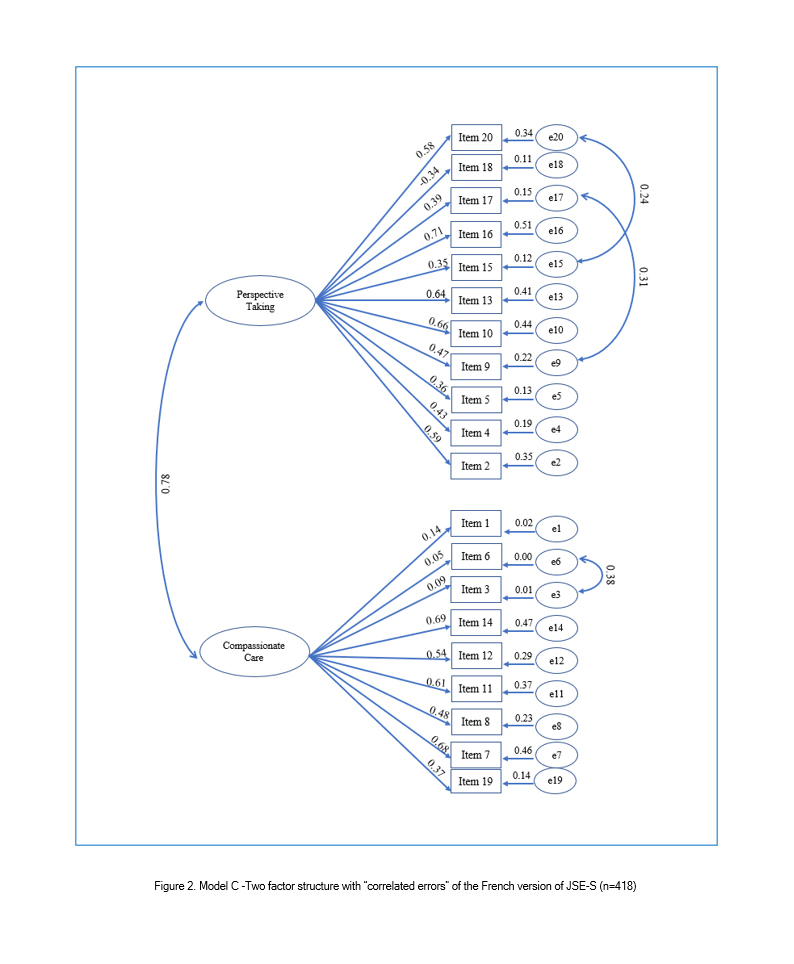
Model C -Two factor structure with “correlated errors” of the French version of JSE-S (n=418)

A review of the modification indices recommends that covariance between six items be allowed: item 3 "It is difficult for a physician to view things from patients' perspectives", item 6 "Because people are different, it is difficult to see things from patients' perspectives",  item 9 "Healthcare providers should try to stand in their patient's shoes when providing care to them", item 15 "Empathy is a therapeutic skill without which a healthcare provider's success is limited", item 17 "Healthcare providers should try to think like their patients in order to render better care", and item 20" I believe that empathy is an important therapeutic factor in medical treatment". With these paths being constrained, the improved fit was produced in each index. This was model C, the two-factor model with "correlated errors" (Table 2, Figure 2). Standardized regression weights and individual items' reliability for models B and C are shown in Figures 1 and 2.

### Known group validity

The mean score of the empathy scale was 104.28 (SD = 15.45) for the whole sample. This score ranged between 34 and 136. Regarding known-group validity (female vs. male) and as expected, there was a significant effect for gender (t _(830)_ = - 4.16, p<.001), with female students receiving higher empathy scores (M = 105.72, SD =14.78) than male students (M = 100.88, SD = 16.50).

## Discussion

The aim of this study was to assess the construct validity and reliability of a French version of JSE-S among medical students. The results of this study supported the previously-reported findings on reliability (Cronbach's α). However, a different construct of the JSE-S has emerged.

Reliability was supported by the satisfactory internal consistency of the scale. The Cronbach's alpha coefficient was 0.78. This value was higher than the one reported for the French version of the JSE-S designed by du Vaure and colleagues (α = 0.65)^17^ and the one reported among Mexican and Iranian medical students (α = 0.74).^20^ However, this value was slightly lower than that reported in other translated versions,^12^^,^^19^^,^^23^^,^^24^ among which the original American version had a value of 0.80.^5^

The construct validity of this French version of JSP-S through principal axing factoring suggested a two-factor solution, namely "Perspective Taking" (PT) and "Compassionate Care" (CC), accounting for 27.4% of the variance. This structure was different from that of the original version and several translated versions;^1^^,^^8^ however, it was similar to that obtained in previous research conducted among students from other healthcare professions using the JSE-HPS-Version. In fact, in Australia, Williams and colleagues found a two-factor solution (PP and CC, Total variance = 44.2%) in 330 paramedic students,^13^ and Fjortoft and colleagues also reported a two-factor solution in 187 first-year pharmacy students (PP and CC, Total variance = 39%).^14^

Thus, the low explained variance reported in this study was less than the accepted 50% for health care psychometrics,^13^ suggesting a potential for another unaccounted variance. Removal of the third factor ("Standing in the Patient's shoes") can be explained by its trivial structure having only two items (Items 3 and 6), as it was described by the designers of this scale.^5^ In fact, this factor may be considered as a residual factor because a minimum of three items per factor is required for a stable factor structure.^1^

Nonetheless, the pattern of the factor structure of this French version of the JSE-S is still somewhat similar to the one found in the original version of the scale. A similar grand factor (perspective taking) also emerged in other translated versions of JSE-S. For instance, in this study, there were ten items under factor 1 and five items under factor 2, also emerging under factors 1 and 2, respectively, in a sample of American medical students.^1^ This statement suggests that despite the modifications made to JSE-S, the underlying components of the scale, particularly the prominent factors of perspective taking and compassionate care, remained intact.^14^ These two factors have been described as the pillars of empathic engagement in patient care.

Our results showed that in addition to items 3 and 6, item 1 ("Physicians' understanding of their patients' feelings and the feelings of their patients' families does not influence medical or surgical treatment"), item 18 ("Physicians should not allow themselves to be influenced by strong personal bonds between their patients and their family members"), and item 19 ("I do not enjoy reading non-medical literature or the arts") yielded low factor loading (r = 0.19, -0.29, 0.23, respectively). This pattern was partially similar to the ones reported among other medical students in Brazil^4^ (item 1; r = 0.30 and item 18; r = 0.34), Korea^28^ (item 18; r = - 0.12 and item 19; r = 0.35), and Iran (items 18 and 19; r < 0.45).^20^ It is, therefore, necessary to revise these items. Overall, their negative wordings and their reverse scoring could be the cause for their low factor loading.^24^ Focusing more on items 1 and 18, low discriminatory values can be attributed to the family-centric approach in medical decision-making. This approach is prevalent in Tunisian society, where individuals are always subordinate to the family or group. However, the low score for item 19 suggests a lower interest of Tunisian students in literature and arts. This can be explained by the admission process to the Tunisian medical schools, which is heavily science-oriented. As a consequence, this process incites school students to focus more on science subjects at the expense of literature and arts. Indeed, further studies are needed to explain these facts.

Despite their low factor loading, the previous five items (1, 3, 6, 18, and 19) were not excluded in the confirmatory factor analysis. In fact, many authors suggest retaining all the items in the scale for comparative purposes, especially since significant item-total score correlations have been reported in most of the psychometric studies of JSE, implying that each item significantly contributes to the total score of the JSE.^6^^,^^14^

The results of the first CFA (Model A) involving the whole sample supported the three-factor model of JSE-S and were in agreement with those reported for the original scale and many translated versions.^5^^,^^6^^,^^8^ However, although modelling of the exploratory solution (two-factor structure) also yielded a good model fit with item correlated errors (Model C), many researchers testing the two-factor model of JSE-S concluded that the three-factor structure is still more appropriate.^1^^,^^6^

Our findings with regard to female medical students obtaining a significantly higher average empathy score than their male counterparts are consistent with previous findings involving American, Mexican, and Japanese^1^^,^^11^^,^^23^ medical students. This statement may also indicate the validity of this French version of JSE-S according to the "contrasted groups" method. It confirms the differences in the expected groups.^14^ However, several studies, including those conducted among Iranian^20^ or Brazilian^4^ medical students, have failed to support this gender hypothesis. The gender difference in empathy has been attributed to intrinsic factors, such as genetics and brain networks, as well as extrinsic factors, including interpersonal style in caring, socialization, and gender role expectation.^4^^,^^23^^,^^26^ However, to highlight this fact, more neuroscience surveys should be conducted. Furthermore, although this study and previous other studies have shown that females have higher scores on empathy scales compared to males, no difference in empathy has been demonstrated between genders in real life settings. For this reason, qualitative studies on empathy in real-life conditions based on audio-or videotaped patient encounters can further explore if a real difference exists.^20^

### Strengths and limitations

This study aimed to analyze the psychometric properties of a French version of JSE-S through both exploratory and confirmatory analyses, as suggested in the literature.^22^ Solid conclusions about the scale dimensionality were endorsed. In addition, this study can be considered informative because different datasets were used to re-examine and, therefore, confirm the factor structure. A split sample of Tunisian medical students was hence involved.

The sample size included in the analysis was more than seven times the number of items and  ≥ 100.^9^ Thus, the overall quality assessment of the structural validity box of COSMIN guidelines can be rated as "very good ".^8^^,^^9^ However, for exploratory factor analysis, principal factoring axing extraction method with oblique rotation was used instead of principal component analysis (PCA), which is the most frequently applied extraction method reported in the literature.^27^ In fact, PFA is more appropriate to uncover the underlying factorial structure of the construct of interest. Yet, PCA is only a data reduction procedure. Moreover, PCA does not separate specific variance and error variance. It often inflates factor loadings and gives an approximate estimation of the factor structure. PFA has the advantage of overcoming this issue attached to PCA and it analyses only specific variance.^28^^,^^29^

Nevertheless, this study has some methodological limitations that should be considered when interpreting the findings. Thus, our results may not be totally generalized because only students from one of the four medical colleges in the country were included, and taking into account the small differences in the curriculum that may influence the levels and understandings of empathy across these colleges. Although we were unable to reach all student cohorts, exposing us to a potential sampling bias, our response rate was high, which may have moderated this limitation. Furthermore, since our findings were based on a cross-sectional design, some important aspects of reliability and validity, such as responsiveness to changes, could not be carried out. In addition, we did not test the current validity of JSE-S with other measures of empathy. Finally, the self-reporting scale of empathy we employed has been reported to be reliable and valid; yet, it only measures medical students' orientation to empathy and not their behavior. A research conducted by Hojat and colleagues, however, demonstrated a correlation supporting a predictive value of JSE-S for empathic behavior.^26^

## Conclusions

The French version of JSE-S used in this study is a psychometrically sound instrument to measure empathy. It can be used as part of the assessment and implementation of empathy among French-speaking medical students. Further research is required to find out whether the reform in medical curricula with a view to train medical students to the highest international standards has a positive impact on students' empathy.

### Acknowledgements

We would like to thank Jefferson Medical College in Philadelphia for permitting the use of the instrument in our research. We would also like to thank the faculty members involved in this study for their help in data collection.

### Conflict of Interest

The authors declare that they have no conflict of interest.
